# A proposed method to investigate reliability throughout a questionnaire

**DOI:** 10.1186/1471-2288-11-137

**Published:** 2011-10-05

**Authors:** Tore Wentzel-Larsen, Tone M Norekvål, Bjørg Ulvik, Ottar Nygård, Are H Pripp

**Affiliations:** 1 Unit of Biostatistics and Epidemiology, Oslo University Hospital, Kirkeveien 116, 0450 Oslo, Norway; 2 Centre for Child and Adolescent Mental Health, Eastern and Southern Norway, Oslo, Norway; 3 Norwegian Centre for Violence and Traumatic Stress Studies, Oslo, Norway; 4 Centre for Clinical Research, Haukeland University Hospital, Bergen, Norway; 5 Department of Heart Disease, Haukeland University Hospital, Bergen, Norway; 6 Faculty of Health and Social Sciences, Bergen University College, Bergen, Norway; 7 Institute of Medicine, University of Bergen, Bergen, Norway

## Abstract

**Background:**

Questionnaires are used extensively in medical and health care research and depend on validity and reliability. However, participants may differ in interest and awareness throughout long questionnaires, which can affect reliability of their answers. A method is proposed for "screening" of systematic change in random error, which could assess changed reliability of answers.

**Methods:**

A simulation study was conducted to explore whether systematic change in reliability, expressed as changed random error, could be assessed using unsupervised classification of subjects by cluster analysis (CA) and estimation of intraclass correlation coefficient (ICC). The method was also applied on a clinical dataset from 753 cardiac patients using the Jalowiec Coping Scale.

**Results:**

The simulation study showed a relationship between the systematic change in random error throughout a questionnaire and the slope between the estimated ICC for subjects classified by CA and successive items in a questionnaire. This slope was proposed as an awareness measure - to assessing if respondents provide only a random answer or one based on a substantial cognitive effort. Scales from different factor structures of Jalowiec Coping Scale had different effect on this awareness measure.

**Conclusions:**

Even though assumptions in the simulation study might be limited compared to real datasets, the approach is promising for assessing systematic change in reliability throughout long questionnaires. Results from a clinical dataset indicated that the awareness measure differed between scales.

## Background

Questionnaires are used extensively in medical and health care research [1]. To be useful as instruments, they depend on both validity and reliability. Validity is the degree to which a scale measures what it is intended to measure. For example, a question on depression should measure depression and not something more or less related. Reliability on the other hand refers to the stability of a measurement, i.e. to what degree the same results occur on separate occasions. It assesses stability, internal consistency and equivalence. An instrument or questionnaire can be reliable without being valid. Thus, an incorrectly calibrated instrument may yield highly repeatable measurements, but still incorrect values [2,3].

Motivation and interest of participants in clinical studies can differ and thereby their focus on accurately answering questions. If the questionnaires contain many items and require a lot of time to complete, concentration and enthusiasm may change throughout the questionnaire. Krosnick [4] outlined how respondents deal with the substantial effort of answering surveys and the effect of satisficing. Satisficing occurs when respondents only give a satisfactory answer instead of spending the mental effort necessary to give optimal answers to question after question. One of the forms of satisficing, caused by distracted, tired or unenthusiastic participants, is when the response alternatives are randomly chosen. Reliability is concerned with random error, i.e. how consistently a scale measures what is it supposed to measure. If satisficing during long surveys and questionnaires increases random error, reliability will be poorer compared to answers by motivated and concentrated participants. Concentration of participants, and thereby possibly random error and reliability, could differ throughout the answering process.

Reliability can be assessed in different ways; test-retest reliability for stability, inter-item reliability for internal consistency and interrater reliability or parallel scale for equivalence [5]. The intraclass correlation coefficient (ICC), i.e. the proportion of the total variability that is explained by the variation between subjects, and Cronbach's alpha coefficient for internal consistency are common statistics on reliability [6]. However, it is not always possible to use these measures due to lack of data or characteristics of the questionnaire. New methods to assess reliability and change in random error due to satisficing are needed.

Subjects in clinical studies can be assigned to subsets by cluster analysis. The similarity between subjects within a subset should be higher than between subsets. However, with increased random error, similarity within subsets can be reduced compared to data from different subsets. Several methods for cluster analysis are available and results from questionnaires are suitable for dividing participants into subsets.

The objective of this study was therefore to simulate the relationship between systematic changes in reliability, expressed as proportion of random error, throughout a questionnaire and how it can be detected. Our proposed method is to divide respondents into subsets using cluster analysis on questionnaire items. The ICC is then estimated for each item based on a mixed effects model. The slope between ICC and item number is proposed as an awareness measure. If this approach can assess systematic change in reliability throughout a questionnaire, it may have an applied potential in "screening" questionnaires on reliability properties. The approach will also be explored using a clinical dataset.

## Methods

### The awareness measure

It was assumed that a number of subjects (n) answered a given number of questions (q), measuring a single construct. These q questions usually comprise part of a questionnaire, often with other questions in between. The subjects were divided into two subsets using cluster analysis (CA). Next, a mixed effects model with no covariates (fixed effect as intercept only) and a random between group effect was run for each item t of the q questions, with subset (from CA) as grouping factor. From this mixed effects model an intraclass correlation coefficient ICC_t _was computed for each item t, as

$$ICC_{t}=\frac{\sigma_{between,t}^{2}}{\sigma_{between,t}^{2}+\sigma_{within,t}^{2}}.$$

**Figure 1:**
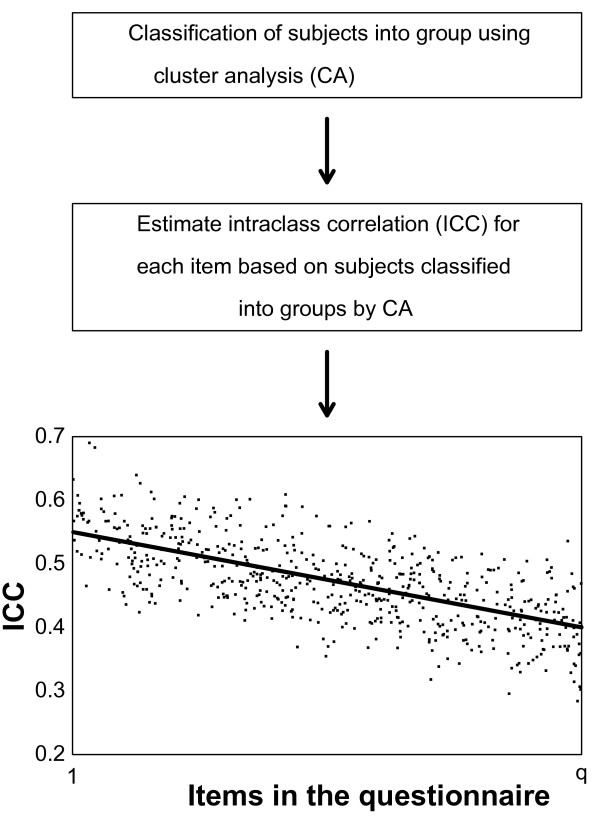
**Proposed method to detect change in reliability throughout questionnaires**. The flow chart shows the steps of the proposed method. The slope between ICC and item number is proposed as a measure to detect change in reliability. A negative slope indicates increased random error and poorer reliability and a positive slope indicates decreased random error and improved reliability. This slope is our awareness measure (see Table 1 and Figure 3)

Finally, a linear regression on intraclass correlation by item number was done. If the cluster analysis or the mixed effects models did not converge for some items, the linear regression was based on the remaining items. The slope from this linear regression was the awareness measure throughout questionnaires, with a negative slope indicating reduced reliability towards the end of the questionnaire due to increased random error or a positive slope indicating increased reliability towards the end of the questionnaire due to reduced random error. An illustration of the procedure is shown in Figure 1.

When the procedure was used with real datasets, confidence intervals of the slope were based on 10000 bootstrap replicates. The slope was considered as significantly different from zero if zero was outside a 95% bootstrap BC_a _confidence interval [7]. Our assumption was that the ICC estimated from the unsupervised clustering of subjects from CA would on average change for each item, if the random error changed systematically for successive items. The slope between ICC and item number was thus proposed as a statistic to detect systematic change in random error, and hence changed reliability. Such a scenario could be the case if participants get more fatigued and unmotivated resulting in more satisficing towards the end of long questionnaires, and therefore have a poorer reproducibility and increased random error towards the last questions compared to the first ones.

### Simulation study

**Figure 2:**
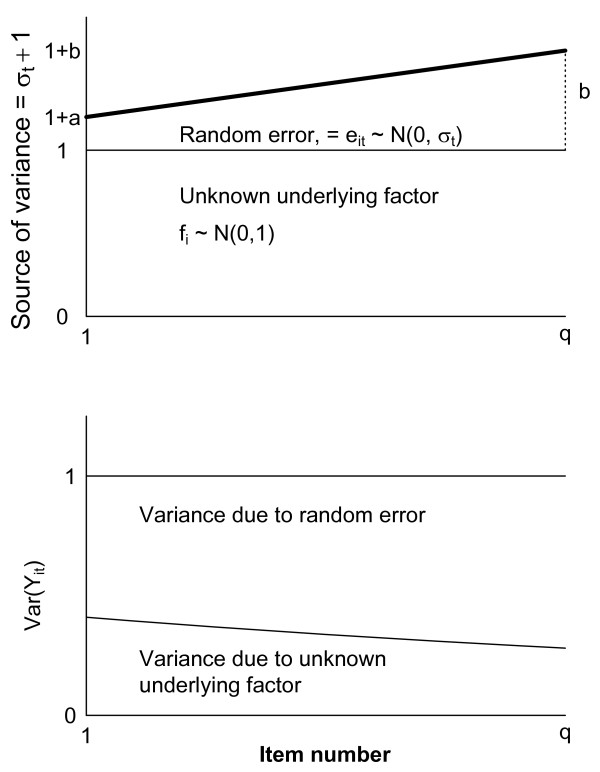
**Assumptions in the simulation study**. The sources of variance are assumed due to an underlying factor (f_i_) and random error (e_it_). Increased random error represents poorer reliability. Total variance, Var(Y_it_), is standardized to 1.0 before estimation of the awareness measure.

The simulation study was conducted to investigate a systematic change in random error, and hence changed reliability throughout a questionnaire. A basic assumption was the existence of an unknown underlying factor (f_i_) partially determining the questionnaire items considered. A unique source of variance (e_it_) was included which was assumed to systematically increase or decrease throughout the scale, reflecting changed reliability. Specifically, we assumed that the answer (y_it_), for each person i on each item t was given by

$$y_{it}=\left(f_{i}+e_{it}\right)∕\sqrt{1+\sigma_{t}^{2}},$$

where

$$\begin{array}{c} \sigma_{t}=a+\left(b-a\right)\frac{t-t_{f}}{t_{l}-t_{f}},\\ f_{i}\sim N\left(0,1\right),\\ e_{it}\sim N\left(0,\sigma_{t}^{2}\right), \end{array}$$

and all f_i _and e_it _are independent from the first (t_f_) to the last (t_l_) items used within the scale with fixed numbers of a and b. Division by $\sqrt{1+\sigma_{t}^{2}}$ ensured that all items had the same variance throughout the simulation (Figure 2).

The awareness measure was computed as indicated above, and its distribution in 10000 simulations reported for different scenarios determined by chosen values of a, b and number of questions (q). For q we used 4 or 13 items, evenly distributed among 50 questions, and the number of persons was 600. 1) A negative value of the awareness measure was expected if a was considerably less than b, simulating the scenario with decreasing awareness throughout the questionnaire. 2) An awareness measure close to 0 was expected if a was approximately equal to b, simulating the scenario with constant awareness throughout the questionnaire. 3) A positive value of the awareness measure was expected if a was considerably larger than b, simulating the scenario with increased awareness throughout the questionnaire (Figure 2).

### Patient sample

The clinical dataset was from a study conducted between August 2000 and February 2002. The source population included 1283 patients admitted to elective coronary angiography at the Department of Heart Disease, Haukeland University Hospital, Bergen, Norway. At least 214 of these patients were not invited to participate due to capacity reasons. Among the remaining 1069 eligible patients, 753 patients (70%) responded. However, due to missing items, 632 individuals with valid values constituted the study population [8]. Ethical recommendation was obtained from the Regional Committee of Medical Research Ethics, Norway. The participants gave written informed consent after having received written information about the study. Most demographic and clinical variables were included in a questionnaire delivered shortly after inclusion, while a second questionnaire including the Jalowiec Coping Scale (JCS) followed by the Hospital Anxiety and Depression Scale (HADS) was handed out a few days later. Only JCS items are used in our study.

### The Jalowiec Coping Scale

The revised 60 items Norwegian version of JCS was used [9-11]. The respondents were asked to rate how often they used each of the 60 coping strategies on a fourpoint scale from 0 (never used) to 3 (often used). A possible total score ranging from 0 to 180 can be calculated. The following sub-scales have shown satisfactory internal consistency in most studies, including the study originally using this dataset [8], and were used for the awareness measure: Confrontive, Evasive, Optimistic and Self-Reliant coping. Since the factor structure of JCS has been discussed [8], we also used the following scales from an alternative factor structure suggested by Wahl et al. [12]: Confrontive, Normalizing optimistic and Combined emotive. The items in all these seven scales are scattered throughout the 60 items in the JCS questionnaire. An overview of number of items and subjects responding to each sub-scale is given in Table 1.

**Table 1:** Estimated awareness measure (AM) in a clinical dataset using the Jalowiec Coping Scale.

Scale	# Items	Resp.	N	AM (95% BC_a_)
Confrontive^a^	10	639	563	0.0030 (-0.0002, 0.0096)
Evasive^a^	13	642	541	0.0020 (0.0012, 0.0057)
Optimistic^a^	9	643	577	0.0077 (0.0059, 0.0115)
Self-reliant^a^	7	637	573	0.0001 (-0.0051, 0.0059)
Confrontive^b^	12	642	549	0.0018 (-0.0026, 0.0038)
Normalising optimistic^b^	10	640	582	0.0098 (0.0055, 0.0138)
Combined emotive^b^	9	646	590	-0.0038 (-0.0069, -0.0004)

# Items: Number of items in selected factor structure. Resp.: Number of the 753 respondents completing at least one item in the scale. N: Number of respondents completing all items in the scale - the analyses are based on these. AM: Awareness measure.

^a ^Jalowiec's original scale.

^b ^Wahl's alternative factor structure [12].

### Statistical software

All computations were conducted with the software R version 2.9.1-2.12 (The R Foundation for Statistical Computing, Vienna, Austria), with the R packages cluster with the clara function for cluster analysis [13], nlme for mixed effect models [14] and boot [15] for bootstrapping.

## Results

### Simulation study

**Figure 3:**
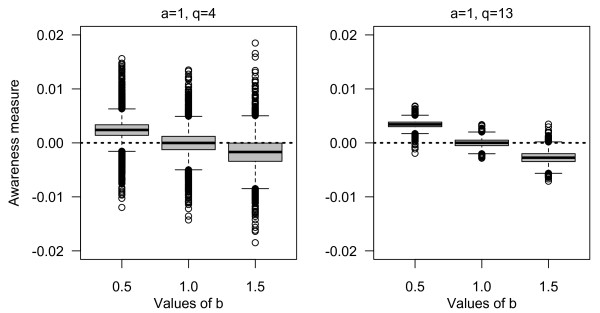
**Results from the simulation study**. The boxplots of estimated awareness measures from simulated data to detect change in reliability are expressed as random error. Number of simulations for each set of condition is 10000. Estimations of the awareness measure are done under scenarios with different values of a, b and q (see Figure 2). The number of subjects is 600.

The boxplots from 10000 simulations of the proposed awareness measure to explore reliability or different scenarios is presented in Figure 3. If random errors are equal throughout the questionnaire (i.e. a and b equal), the mean value of the estimated slope is approximately zero. The slope coefficient is more negative with increased random error throughout the questionnaire (a considerably less than b) or more positive with decreased random error throughout the questionnaire (a considerably larger than b). However, the effect on the awareness measure was larger for shorter questionnaires (i.e. q = 4 compared with q = 13) given the same scenario conditions.

### Clinical dataset on JCS

The results are shown in Table 1. The most positive slope is 0.0098 (95% BC_a _0.0055, 0.0138) for the Normalising optimistic scale, whilst the Optimistic scale is somewhat lower, 0.0077 (95% BC_a _0.0059, 0.0115). Thus, based on our proposed method, awareness and thereby reliability increased throughout the answering process for both optimistic scales. In contrast, the slope for the Combined emotive scale is negative, -0.0038 (95% BC_a _-0.0069, -0.0004). Based on our proposed method, reliability decreased throughout the questionnaire for the Combined emotive scale.

## Discussion

The simulations showed that our procedure is able to detect changes in awareness, if such results in changed random error, during completion of a questionnaire for a single factor scale. When the procedure is used on a real data set, the results depend on the scale. The highest slope (in absolute value) is 0.0098 per question for the Normalising optimistic scale, corresponding to an increase of 0.0098⋅60 = 0.59, with confidence limits 0.33 and 0.83, during the 60 JCS questions. The slope for the Optimistic scale is somewhat lower. In contrast to this, the negative slope -0.0038 for the Combined emotive scale corresponds to -0.0038⋅60 = -0.23, with confidence limits -0.41 and -0.02 during 60 questions. These slopes are large enough to indicate relevant changes during the 60 items JCS questionnaire on reliability according to our proposed method. For the other scales there were smaller changes. It is interesting that the two optimistic scales with items for "positive" and constructive coping strategies also have increased reliability throughout the scale according to the proposed awareness measure. Perhaps the participants want to emphasis and answer very accurately on these items? Thus, confirming "positive" coping strategies. The Combined emotive scale contained many "negative" coping strategies like e.g. "avoided being with people", "took your tensions on someone else" and "took medications to reduce tensions". A hypothesis is that participant may feel embarrassed or hopeless about using such strategies, and thus, do not want to answer accurately. Both these tendencies may increase as the participants become accustomed to answering coping questions.

Reliability and reproducibility are crucial in all quantitative research. Diagnostic tests are conducted under supervised conditions to assure both validity and reliability of results. Laboratory analyses in areas as genetics, chemistry or physics are often done in replicates to assess reliability. Likewise, reliability of questionnaires and surveys should be assessed when used as measurement tools in research. Predictions from statistical models are limited by reliability of measurements [16]. It should therefore be useful to employ "screening" methods for assessment of reliability throughout lengthy questionnaires.

The aim for our proposed method is to serve as a screening test to detect reliability differences throughout a scale. Questionnaire length may reduce the motivation of the participants. A meta-analysis on questionnaire length showed lower response rate on long compared to shorter questionnaires [17]. Low response rate may thereby induce bias. Galesic & Bosnjak [18] found that fewer respondents started and completed questionnaires with longer stated length. Answers to questions positioned later in the questionnaire were shorter and more uniform than answers to questions positioned at the beginning. In this study, we hypothesized that the satisficing effect would give increased random error throughout the questionnaire (Figure 1) resulting in a negative slope between estimated ICC and item number after CA (Figure 2). However, results from the clinical dataset showed that it might be different for different scales representing underlying factors. Our results suggest that items that indicate attractive and positive coping strategies are answered with increased reliability (Table 1).

Item Response Theory (IRT) (see e.g. textbooks by Lord [19] or De Alaya [20] for a thorough explanation) might also be a promising approach to investigate changed reliability during long questionnaires. IRT is used frequently on measurement of ability and achievement, but so far less in clinical assessment [21]. In Classical Test Theory (CTT) a test score has the same meaning independent of the subjects(s) of assessment, e.g. length has the same meaning whether measuring an arm or a foot, while IRT develops models where characteristics of examinee and tests can be separated. Its key element is the assumption that the probability of a subject's answer to an item is a function of two sets of parameters: 1) their standing on the latent variable of interest (e.g. IQ) - the person parameter; and 2) the characteristics of the item (e.g. severity of the question) - the item parameter. The concept of information from IRT is inversely related to standard error of measurement. In traditional CTT, reliability is assumed to be constant for all subjects, but information from IRT is allowed to be different between subjects. This could be explored in relation to different awareness among subjects during lengthy questionnaires. Perhaps the most promising application of IRT is development of computer adaptive tests (CATs). The principle is to provide estimates of subjects standing on latent variables, e.g. depression, and choose subsequent items in a manner that will maximize information. It can significantly reduce the number of questions needed as well as test time. In a clinical setting, the burden to patients undergoing a CAT is substantially reduced because patients need to answer only questions with particular relevance to their own individual situation [22].

There are several assumptions in our simulation study. Scales are assumed to represent a uniform underlying factor for all participants. Our mixed results on scales in the real data set may be due to problems with the factor structure. However, the scales used have acceptable internal consistency in most studies, and we have used scales from two alternative factor structures, with similar results. The mixed results may also indicate that changes in awareness during a long questionnaire constitute a more complex process, depending on context and the individual items. Satisficing can also take other forms than randomly answering items and be a more systematic process [23]. Other effects due to satisficing could influence the results from our proposed awareness measure. Thus, the proposed awareness measure may help assess changed reliability, but not give a complete assessment of satisficing.

## Conclusions

An awareness measure was proposed to explore changes in reliability throughout questionnaires. The simulation study showed that the systematic change in random error was detected by estimating the ICC between subjects unsupervised classified by CA. In the real data set, however, different changes were observed for different scales.

Response burden always needs to be considered when planning a study. Consequently, when applying long questionnaires, reliability should be evaluated. We suggest using CA and estimation of ICC to assess potential systematic change in reliability.

## Competing interests

The authors declare that they have no competing interests.

## Authors' contributions

TWL carried out the simulations and drafted the manuscript. AHP developed the initial idea for the work, participated with simulations and drafted the manuscript. TMN, BU and ON participated in the preparation of data and selection of scales for investigation. All authors read and approved the final manuscript.

## Pre-publication history

The pre-publication history for this paper can be accessed here:

http://www.biomedcentral.com/1471-2288/11/137/prepub
